# Norovirus Infection in Children with Acute Gastroenteritis, Madagascar, 2004–2005

**DOI:** 10.3201/eid1306.070215

**Published:** 2007-06

**Authors:** Dimitrios C. Papaventsis, Winifred Dove, Nigel A. Cunliffe, Osamu Nakagomi, Patrice Combe, Pierre Grosjean, C. Anthony Hart

**Affiliations:** * University of Liverpool, Liverpool, United Kingdom; † Nagasaki University, Nagasaki, Japan; ‡ Institute Pasteur, Antananarivo, Madagascar

**Keywords:** Calicivirus, norovirus, genogroup, gastroenteritis, child, Madagascar, research

## Abstract

Of 237 children with acute gastroenteritis in Antananarivo, Madagascar, during May 2004–May 2005, 14 (≈6%) were infected with norovirus. Seasonality (November–December peak) was detected. Reverse transcription–PCR identified GII as the most common genogroup. GIs belonged to GI.1, GI.3, and GI.4. Noroviruses in Madagascar show extensive genetic diversity.

Gastroenteritis is a major public health issue worldwide. Noroviruses are now considered emerging pathogens (*1*) and are recognized as the leading cause of nonbacterial, acute gastroenteritis in humans (*2*). The 2 genera, *Norovirus* and *Sapovirus,* are members of the family *Caliciviridae* and have a positive-sense, single-stranded RNA genome ≈7.5 kb long. Because no readily available cell culture system exists, characterization and classification of noroviruses are based on reverse transcription (RT)–PCR, genomic sequencing, and phylogenetic analysis (*2*–*5*). According to the latest scheme for norovirus nomenclature, the 29 genetic norovirus clusters or genotypes are classified into 5 genogroups (GI–V) (*6*). GI and II infect humans, and GII.4 has been the most highly prevalent genotype worldwide during the past decade (*2*,*5*,*7*).

The saying “Madagascar is not an island, but an archipelago” captures an important aspect of the country, whose geography and history have combined to produce a society of considerable diversity and uniqueness (*8*). In recent years, many studies have investigated the role of human noroviruses in childhood diarrhea and found worldwide distribution (*4*,*7*,*9*,*10*). However, no studies have reported the prevalence and molecular epidemiology of noroviruses in Madagascar, which we report here.

## The Study

From May 2004 to May 2005, a study of acute gastroenteritis in children <16 years of age was undertaken by the Institut Pasteur, Antananarivo, Madagascar. Children with a diagnosis of acute dehydrating watery diarrhea who were seen at the rehydration clinics and hospitals of Antananarivo were eligible for the study. Antananarivo, the capital city of Madagascar, has a population of ≈4 million. The study was approved by the Ethical Review Board of the Institut Pasteur, Antananarivo, Madagascar.

Fecal samples were collected from the children and stored at –80°C until analysis was undertaken at the University of Liverpool, UK. There, viral RNA was extracted from 150 μL of 10%–20% fecal suspensions in phosphate-buffered saline by using a guanidine and silica method (*11*). Sapoviruses were not sought in the initial screening. RT-PCR was performed in a 50-μL reaction mixture with primers that targeted the capsid N terminus/shell gene. For GI, forward primer G1SKF (5′-CTGCCCGAATTYGTAAATGA-3′) and reverse primer G1SKR (5′-CCAACCCARCCATTRTACA-3′) were used, yielding a PCR product of 330 bp. For GII, the primers used were forward primer G2SKF (5′-CNTGGGAGGGCGATCGCAA-3′) and reverse primers G2SKR (5′-CCRCCNGCATRHCCRTTRTACAT-3′) and G2ALSKR (5′-CCACCAGCATATGAATTGTACAT-3′), yielding a 344-bp product (*12*). The PCR was performed with an initial denaturation at 94°C, followed by 40 cycles of 60-sec denaturation at 94°C, 60-s primer annealing at 50°C, an extension for 2 min at 72°C, followed by a final extension stage of 15 min at 72°C. Amplification products were examined under ultraviolet light after electrophoresis through a 2% agarose gel with ethidium bromide staining. Products were extracted by using the QIAquick PCR purification kit (QIAGEN, Basingstoke, UK) and were sequenced by Macrogen Inc. (Seoul, South Korea).

Phylogenetic relationships were examined by aligning sequences with the ClustalW multiple alignment program (European Molecular Biology Laboratory, Heidelberg, Germany). A phylogenetic tree was constructed according to the neighbor-joining method by using ClustalX (version 1.83) and the alignment file obtained by analysis with ClustalW. Bootstrap values on a scale from 1 to 1,000 were also calculated. An unrooted phylogram of norovirus isolates from the present study and prototype strains was plotted in the PHYLIP format (http://evolution.genetics.washington.edu/phylip.html) output by using TreeView software, version 3.1 (http://taxonomy.zoology.gla.ac.uk/rod/treeview.html). Assignment of norovirus to genotype was made according to the scheme proposed by Zheng et al. (*6*). The nucleotide sequences of the Malagasy strains have been deposited at GenBank (accession nos. EF213624–EF213635, EF213638, and EF213640.) Norovirus-positive samples were screened for other viruses by negative-stain electron microsopy and by RT-PCR for rotavirus and astrovirus (*13*,*14*).

During this 12-month study, 258 children with acute gastroenteritis in Madagascar were screened for norovirus infection. Because 21 samples contained insufficient stool, 237 samples were analyzed (142 from boys and 95 from girls). Overall, 85% of children were <3 years of age, 77% were <2 years, 43% were <1 year, and 3% were newborns. The median age of the study population was 20 months (range 1 day to 16 years).

**Figure 1:**
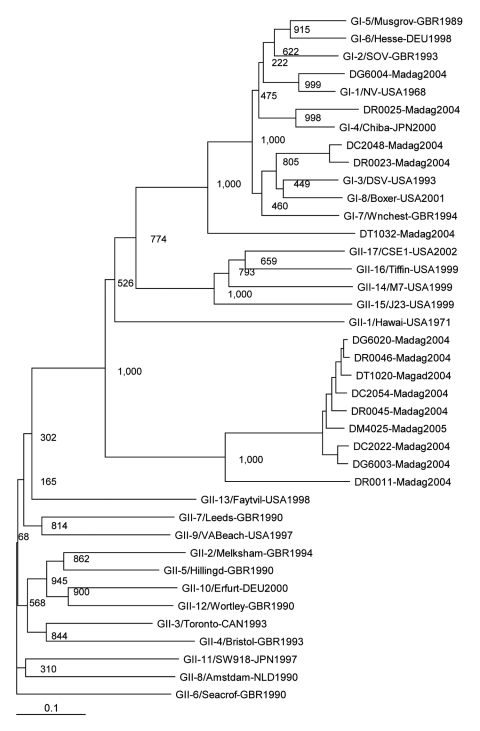
Phylogenetic tree of noroviruses based on the 330-bp region (for GI) and 344-bp region (for GII) of the capsid N terminus/shell gene. Fourteen novel sequences were included, designated according to isolate code, place, and year (e.g., DR001-Madag04); 25 sequences of reference norovirus strains (*6*) were included, designated according to genogroup-genotype, place, country, and year (e.g.,GII-2/Melksham-GRB1994). Comparative strains are GI-1/NV-USA1968 (Norwalk, M87661), GI-2/SOV-GBR1993 (Southampton, L07418), GI-3/DSV-USA1993 (Desert Shield, U04469), GI-4/Chiba-JPN2000 (AB042808), GI-5/Musgrov-GBR1989 (Musgrove, AJ277614), GI-6/Hesse-DEU1998 (AF093797), GI-7/Wnchest-GBR1994 (Winchester, AJ277609), GI-8/Boxer-USA2001 (AF538679), GII-1/Hawai-USA1971 (U07611), GII-2/Melksham-GBR1994 (X81879), GII-3/Toronto-CAN1993 (U02030), GII-4/Bristol-GBR1993 (X76716), GII-5/Hillingd-GBR1990 (Hillingdon, AJ277607), GII-6/Seacrof-GBR1990 (Seacroft, AJ277620), GII-7/Leeds-GBR1990 (AJ277608), GII-8/Amstdam-NLD1990 (Amsterdam, AF195848), GII-9/VABeach-USA1997 (AY038599), GII-10/Erfurt-DEU2000 (AF427118), GII-11/SW918-JPN1997 (AB074893), GII-12/Wortley-GBR1990 (AJ277618), GII-13/Faytvil-USA1998 (Fayetteville, AY113106), GII-14/M7-USA1999 (AY130761), GII-15/J23-USA1999 (AY130762), GII-16/Tiffin-USA1999 (AY502010), and GII-17/CSE1-USA2002 (AY502009). Bootstrap values based on 1,000 generated trees are displayed at the nodes. The scale bar represents nucleotide substitutions per site.

Fourteen noroviruses (5.9%) were detected in 237 children (Table 1). Noroviruses were found in all age groups. Infection rates did not differ between boys and girls (6.3% and 5.2%, respectively, p>0.1). No coinfections with other viruses (rotavirus, astrovirus, and adenovirus) were detected. Ten (71%) of the noroviruses detected were identified as belonging to genogroup GII; the remaining 4 (29%), to GI. Most GII noroviruses belonged to a potentially novel cluster (Figure 1). GI noroviruses were further classified into 3 genotypes: GI.1 (1 isolate), GI.4 (1 isolate), and GI.3 (2 isolates).

**Table 1:** Characteristics of viruses from children with acute gastroenteritis in Madagascar*

Identification no.	GenBank accession no.	Sample† date	Age, mo	Sex	Norovirus PCR	Norovirus sequencing	EM for NoV, RV, AV‡
DG6004-Madag04	EF213640	May 2004	14	M	GI pos	GI-1	Neg
DC2022-Madag04	EF213626	July 2004	14	F	GII pos	GII novel	Neg
DG6003-Madag04	EF213634	Oct 2004	44	M	GII pos	GII novel	Neg
DR0011-Madag04	EF213628	Nov 2004	12	M	GII pos	GII novel	Neg
DR0025-Madag04	EF213625	Nov 2004	17	M	GI pos	GI-4	Neg
DC2048-Madag04	EF213624	Nov 2004	3	M	GI pos	GI-3	Neg
DR0023-Madag04	EF213638	Nov 2004	9	F	GI pos	GI-3	Neg
DR0045-Madag04	EF213627	Nov 2004	19	F	GII pos	GII novel	Neg
DC2054-Madag04	EF213629	Dec 2004	16	M	GII pos	GII novel	Pos (putative NoV)
DG6020-Madag04	EF213630	Dec 2004	30	M	GII pos	GII novel	Neg
DR0046-Madag04	EF213631	Dec 2004	14	F	GII pos	GII novel	Neg
DT1020-Madag04	EF213632	Dec 2004	12	F	GII pos	GII novel	Neg
DT1032-Madag04	EF213633	Dec 2004	8	M	GII pos	GII novel	Neg
DM4025-Madag05	EF213635	Mar 2005	51	M	GII pos	GII novel	Neg

*EM, electron microscopy, NoV, norovirus; RV, rotavirus; AV, astrovirus; pos, positive; neg, negative. †Fecal samples. ‡All norovirus-positive samples were negative for AV and RV when tested by reverse transcription–PCR.

**Figure 2:**
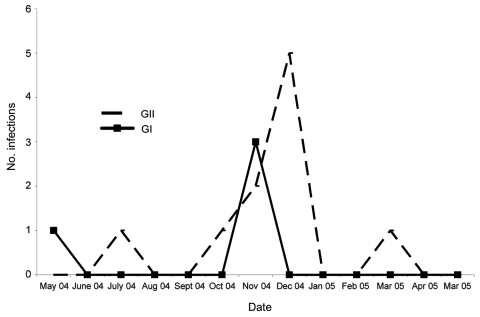
Seasonality of GI and GII norovirus infections, Antananarivo, Madagascar, May 2004­­–May 2005.

The median age of children with norovirus infection was 18 months (range 3–51 months). Most infections (≈86%) occurred in children <36 months of age. No infections were recorded in newborns or children >5 years of age. All infections with genogroup GI noroviruses were found in children <24 months of age (Table 2). Noroviruses were detected throughout the year; however, infections peaked during the wet season in Madagascar. November and December were the months of major norovirus prevalence (35.7% each month). GI noroviruses preceded GII infections by a few days (Figure 2).

**Table 2:** Distribution of viruses and norovirus genogroups in children with acute gastroenteritis, by age, Madagascar, May 2004­­–May 2005

Virus	Age group, mo			
	0–12	13–24	25–36	>36
Other than norovirus	96	75	18	34
Norovirus GI	2	2	0	0
Norovirus GII	3	4	1	2

## Conclusions

To our knowledge, ours is the first study that has used molecular detection methods to investigate the role of noroviruses in pediatric gastroenteritis in Madagascar. We showed that infections with GI and GII noroviruses are relatively common. In a 1-year collection of stool samples, we detected noroviruses by RT-PCR in ≈6% of children with acute gastroenteritis in Antananarivo. This rate establishes norovirus as the second most commonly detected enteric virus in this population, behind rotavirus (38%) and followed by astrovirus (2.5%) (data not shown). These findings are consistent with those of studies elsewhere (*7*,*10*).

The median age of children with norovirus infection (18 months) was higher than previously reported (*7*) and higher than that of the rotavirus-infected group (median 10 months, range 1 day to 48 months) and that of the astrovirus-infected group (median 10 months, range 5–20 months) (data not shown). Noroviruses were detected throughout the year, but the number peaked in November and December. Such seasonality in a tropical country is not really expected, as year-round circulation has been previously documented (*15*).

Our findings confirm the finding of previous studies that GII is the predominant norovirus genogroup circulating in communities worldwide. Considerable genetic diversity was observed among the norovirus GII isolates, and some were identified as belonging to a potentially novel cluster. The closest reference strain to the potentially novel cluster was the recombinant Hu/NoV/GII.1/Hawaii/1971/US. In contrast, norovirus GI isolates were clustered with prototype strains; Hu/NoV/GI.3/DSV395/1990/SA (Desert Shield) was predominant (2 strains), followed by Hu/NoV/GI.1/Norwalk/1968/US (Norwalk) and Hu/NoV/GI.4/Chiba407/1987/JP (1 each).

This study has several limitations. First, it is a preliminary study. The sample size was small, and we examined samples collected over a 13-month period. Longer, longitudinal studies are required to address issues such as norovirus seasonality and temporal genetic variability. In addition, we restricted our analysis to specimens collected from patients at rehydration clinics and hospitals, so prevalence of norovirus infections in the general population may have been underestimated. Furthermore, the use of short conserved sequences, although successful for diagnosis of norovirus infection, should be used with caution for classification and phylogenetic analyses. Further analysis by full capsid sequencing might be required. Nevertheless, continued norovirus surveillance is needed to monitor the spread and persistence of the various genotypes infecting children in Madagascar.
